# Editorial: New perspectives of hypoxic ischemic encephalopathy

**DOI:** 10.3389/fped.2023.1251446

**Published:** 2023-07-14

**Authors:** Mehmet Satar, Emel Okulu, Hacer Yapıcıoğlu Yıldızdaş

**Affiliations:** ^1^ Faculty of Medicine, Department of Pediatrics, Division of Neonatology, Çukurova University, Adana, Turkey; ^2^ Faculty of Medicine, Department of Pediatrics, Division of Neonatology, Ankara University, Ankara, Turkey

**Keywords:** hypoxic ischemic encephalopathy, therapeutic hypothermia, cerebral metabolism, c-reaktive protein, adjuvant therapies

**Editorial on the Research Topic** New perspectives of hypoxic ischemic encephalopathy

Hypoxic ischemic encephalopathy (HIE) is a significant cause of mortality and short- and long-term morbidities. The estimated incidence of HIE is 1.5/1,000 live births (1). However, there are significant discrepancies in reported incidences between population- and hospital—based studies. It is assumed that the range for HIE is about 1–8/1,000 live births (2).

Although hypoxic-ischemic injury of the infant brain may occur both during the antepartum and postnatal periods, it is much less common than the intrapartum period. 5%–20% of neonatal HIE is caused by hypoxic-ischemic injury during the antenatal period, while 56% of all cases with HIE were associated with hypoxic-ischemic insults that occurred during the intrapartum period, as shown in a large population-based observational study (3). It is assumed that intrapartum hypoxic ischemia is more common in developing countries than in developed countries. Postpartum events solely, such as heart failure, severe pulmonary disease, etc., can lead to HIE and may account for approximately 5%–10% of cases (1, 3, 4).

Neurological dysfunction is the case but is the most important of injury following hypoxic-ischemic trauma. Infants may also have coexisting multiorgan dysfunction, which increases the risk of morbidity and mortality. In addition to having a compromised central nervous system, all infants with severe post-asphyxial HIE had evidence of at least one other organ or system dysfunction. Pulmonary, hepatic, renal, and cardiovascular involvement were most commonly reported in studies (O’Dea et al., 5).

Therapeutic hypothermia (TH) has been shown to be the standard care for HIE of infants ≥36 weeks GA, decreasing the rates of mortality, cerebral palsy, hearing and visual impairment, and neurodevelopmental delay. The 2013 Cochrane review analyzed 11 randomized controlled trials including 1,505 infants and found that TH reduced mortality without increasing major disability in survivors (6).

The currently published study assessed the effectiveness of TH for HIE in low-income countries and concluded that it was neither effective nor safe and advised against its use, where these results and conclusion should be questioned (7).

The abnormal outcomes at follow-up were reported in a significant proportion of infants with even mild HIE (8). For infants with mild HIE, there are currently insufficient data that recommend routine TH, but significant advantages or risks cannot be excluded (9). A survey of cooling centers in the United Kingdom showed that 75% of these centers offered TH to infants with mild HIE (10).

The current recommendation is to initiate TH before 6 h of life (6). A recent retrospective, observational cohort study by Guillot et al. showed that early TH started before 3 h of life was not associated with fewer brain lesions on MRI or better neurodevelopmental outcomes (11).

The benefit of TH in preterm infants is still unknown. Azzopardi *et al*. reported that infants who were born at 34 or 35 weeks GA who received TH had a higher mortality rate compared with term infants (12). The two small studies that evaluated the short and long-term outcomes of TH in late preterm infants that TH appears to be feasible in preterm infants but with a concerning incidence of complications and the combined outcome of death and neurodevelopmental outcome in this highly vulnerable population (13, 14).

The goal of this research topic was to update and consolidate the field of biomarkers of neonatal brain injury in preclinical and clinical settings. This includes both circulating and imaging biomarkers, early biomarkers of acute injury, and correlations with long-term neurodevelopment. It is aimed to provide a foundation for future clinical translation and research to guide neuroprotective care in neonatal medicine. In this special e-collection, there are four papers covering some above-mentioned aspects.

One of the papers in this special section was conducted in normothermic and hypothermic piglets. HIE after a perinatal insult is a dynamic process that evolves over time, and the authors investigated early changes in cerebral metabolism after perinatal hypoxia-ischemia under normal and hypothermic conditions. After a standardized hypoxic-ischemic insult, they found the presence of progressive secondary deterioration with concomitant increase in markers of cell lysis as secondary increase in glycerol and extracellular lactate in normothermic piglets. They also found that TH resulted in lower intracerebral pressure, glucose accumulation, and lower levels of extracellular lactate. They concluded that TH treatment appeared to abolish the secondary increase in glycerol concentration Andelius et al.

In another study, the authors evaluated the relationship between TH and whole blood high-sensitivity C-reactive protein (hs-CRP) in neonates with HIE. In this study, they showed that TH can cause noninfectious CRP elevation. TH significantly affects the postnatal course of inflammatory markers, including hs-CRP response and lowers white blood cell and platelet counts. However, there is insufficient evidence that these changes are indicative of infection. Elevated hs-CRP may interfere with the evaluation of infection, leading to inappropriate antibiotic use or prolonged antibiotic courses Wang et al.

Another study in this section is a systematic search of PubMed, Embase, and MEDLINE on the combination of hypothermia and some drugs. The aim was to identify the classes of drugs that have been used in combination with hypothermia for the treatment of neonatal HIE and to determine whether combination therapy is more effective than TH alone. Gamma-aminobutyric acid (GABA) receptor agonists, N-methyl-d-aspartate (NMDA) receptor antagonists, neurogenic and angiogenic agents, stem cells, glucocorticoids, and antioxidants were identified as potential adjuvants to TH. They showed that the length of hospital stay was significantly reduced in infants treated with combination therapy compared with those treated with TH alone, while the risk of mortality and neurodevelopmental impairment did not differ Ovcjak et al.

The final study is a systematic review of Clinical Practice Guidelines (CPGs) for Neonatal Hypoxic-Ischemic Encephalopathy. This systematic review of guidelines addresses neonatal hypoxic-ischemic encephalopathy, which has crucial medical and legal concerns. Appraisal of Guidelines for Research and Evaluation (AGREE) II Instrument was used. Two recent HIE CPGs were eligible and were appraised. These are the Canadian Paediatric Society (CPS) and the Queensland Maternity and Neonatal Services (QMN). The methodological quality of the QMN CPG was superior to the relevant recommendations for its use in neonatal practice. Adaptation of evidence-based national guidelines using the Appraisal of Guidelines for Research and Evaluation (AGREE) II Instrument will benefit society. This systematic review will reduce variation among health care institutions and providers and provide a basis for regulatory authorities to make decisions about optimal neonatal practice and care Amer et al.

Questions that need to be answered about HIE in future studies include the following: There is insufficient evidence to determine whether the use of TH in infants with mild HIE is of significant benefit or harm. What adjuvant therapy in addition to TH provides better outcomes? Could very early initiation of TH affect neurodevelopmental prognosis?
